# Unrecognized high prevalence of expanded composite repeats in Friedreich ataxia

**DOI:** 10.1093/hmg/ddaf190

**Published:** 2025-12-23

**Authors:** Morgan C Devore, Christina Lam, Graham Wiley, Courtney C Park, David R Lynch, Sanjay I Bidichandani

**Affiliations:** Neuroscience Graduate Program, University of Oklahoma Health Sciences Center, 4000 N. Lincoln Blvd., Oklahoma City, OK 73104, United States; Department of Pediatrics, Section of Genetics, University of Oklahoma Health Sciences Center, 1200 Children's Ave., Oklahoma City, OK 73104, United States; Clinical Genomics Center, Oklahoma Medical Research Foundation, 825 NE 13th St., Oklahoma City, OK 73104, United States; Division of Neurology, The Children’s Hospital of Philadelphia, 3615 Civic Center Blvd., Philadelphia, PA 19104, United States; Division of Neurology, The Children’s Hospital of Philadelphia, 3615 Civic Center Blvd., Philadelphia, PA 19104, United States; Neuroscience Graduate Program, University of Oklahoma Health Sciences Center, 4000 N. Lincoln Blvd., Oklahoma City, OK 73104, United States; Department of Pediatrics, Section of Genetics, University of Oklahoma Health Sciences Center, 1200 Children's Ave., Oklahoma City, OK 73104, United States; Department of Biochemistry and Physiology, University of Oklahoma Health Sciences Center, 940 Stanton L. Young Blvd., Oklahoma City, OK 73104, United States

**Keywords:** Friedreich ataxia, proximal *FXN* deletion, genotyping errors, *Alu*-mediated recombination, composite repeat

## Abstract

Many diseases are caused by pathogenic expansion of microsatellite repeats. Longread sequencing allows evaluation of the content of such expanded repeats. Friedreich ataxia patients are typically homozygous for an expanded GAA repeat in intron 1 of the *FXN* gene. Longread whole genome sequencing identified expanded composite alleles, consisting of substantial tracks of tandem GGA triplets within the expanded GAA repeat. In a prospective series of 112 unrelated patients, we found that approximately 20% of people with Friedreich ataxia have at least one such expanded composite allele. Other minor sequence interruptions in the expanded GAA repeat were detected in a further 10% of patients. Most expanded composite alleles revealed by longread genome sequencing are not detectable by standard PCR-based testing, and have therefore remained hidden despite their relatively high prevalence. This results in erroneous genotyping of patients and heterozygous carriers. We describe an optimized workflow to detect these expanded composite alleles, which permitted accurate genotyping and heterozygous carrier identification. A recurrent proximal *FXN* gene deletion caused by *Alu*-mediated non-homologous recombination was identified in an additional 2% of patients. These findings redefine the spectrum of pathogenic alleles in Friedreich ataxia, and demonstrate that expanded alleles containing substantial non-GAA interruptions are prevalent and pathogenic.

## Graphical Abstract

**Graphical Abstract:**
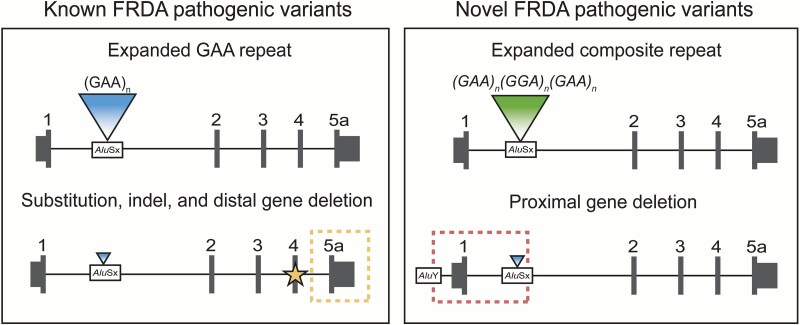

## Introduction

Over 50 genetic diseases are caused by abnormal expansion of microsatellite repeats [1]. Molecular diagnosis is typically established via PCR-based amplification of the repeat-containing region to estimate the length of the expanded tract, or via repeat-primed PCR to determine that the repeat length is greater than the threshold associated with disease pathogenesis. Non-motif sequence variants that may be located deep within the expanded microsatellite repeat tract are thus typically not detected. The availability of longread genome sequencing technologies (e.g. Oxford Nanopore & PacBio sequencing) has made it possible to evaluate the precise sequence of the entire expanded repeat, without PCR amplification, even within very long disease-causing expanded repeats that are typically beyond the purview of standard short-read sequencing technology (e.g. Illumina sequencing). Moreover, longread genome sequencing also has the advantage of revealing local genomic rearrangements (e.g. indels) in the vicinity of the expanded repeat.

Friedreich ataxia, a recessive condition, is typically caused by inheriting an expanded GAA repeat sequence in intron 1 of both copies of the *FXN* gene [2, 3]. Pathogenic alleles, which range from 56 to ~ 1300 triplets (and commonly > 500 triplets) in length, require a ‘long-range’ PCR strategy to detect and size them. In such assays, visualization of two distinct expanded alleles (e.g. 800 and 1000 triplets) and the absence of a normal allele confirms the diagnosis of FRDA. Given that most expanded alleles contain 600–1000 triplets, symptomatic FRDA patients who show only one expanded allele (and absence of a normal allele) are assumed to have inherited expanded alleles of the same (or similar) size (e.g. 800 and 800 triplets). The latter occurs in a substantial proportion (~20%) of FRDA patients, and no further testing is typically performed in the current diagnostic workflow. While detection of a single expanded allele may simply represent inadequate resolution of two expanded alleles of similar size (e.g. 800 and 850 triplets), we hypothesized that in some of these cases we may be missing truly *hemizygous* genotypes, e.g. genomic deletions that preclude amplification of one of the *FXN* alleles. We tested this hypothesis via longread genome sequencing in a cohort of symptomatic FRDA patients with only one identifiable expanded allele, which permitted analysis of the entire *FXN* locus including the entirety of the expanded repeat. This revealed two types of novel pathogenic alleles; expanded composite alleles (most with variable tracks of GGA triplets) and proximal *FXN* gene deletions. Both of these pathogenic alleles have been missed by standard diagnostic testing, which has led to the apparently high prevalence of individuals deemed to be homozygous for identically sized expansions. A large number of pathogenic alleles in Friedreich ataxia have thus remained invisible. This shows for the first time that expanded alleles with substantial non-GAA interruptions are both prevalent and pathogenic in Friedreich ataxia.

## Results

### A substantial number of FRDA patients seem to be homozygous for identically-sized expanded GAA repeats

**Figure 1:**
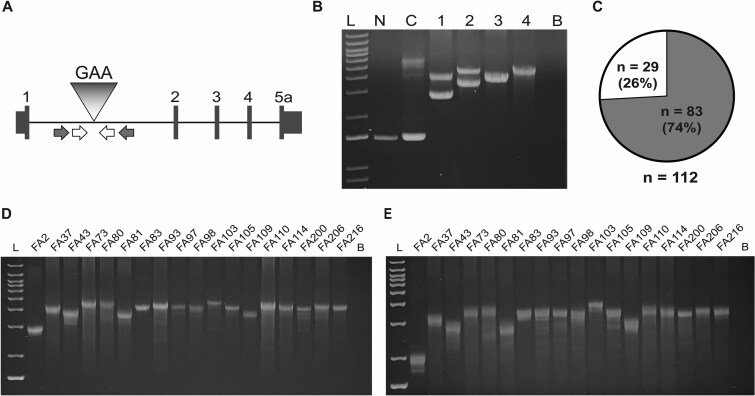
A substantial number of FRDA patients seem to be homozygous for identically-sized expanded GAA repeats. (**A**) Schematic of the *FXN* gene showing coding exons 1–5a, the GAA repeat (GAA), and two primer sets flanking the GAA repeat: Bam & 2500F (gray arrows) and GAA-104F & GAA-629R (white arrows). (**B**) Representative results of a long-range PCR assay showing a non-FRDA control (N), a heterozygous carrier (C), FRDA patients with two distinct expanded alleles, i.e. the ‘double-band’ pattern (1 & 2), and FRDA patients with only one discernible expanded allele, i.e. the ‘single-band’ pattern (3 & 4). L = 1 kb ladder; B = blank (no template) control. (**C**) In a total of 112 unrelated FRDA patients, 26% had the ‘single-band’ pattern. (**D, E**) long-range PCR analysis in 18 FRDA patients (lab IDs are indicated) showing a ‘single-band’ pattern with two different primer combinations. L = 1 kb ladder; B = blank (no template) control.

In a prospective series of 112 unrelated FRDA patients (with a commercial genetic test indicating they are homozygous for expanded GAA repeats in the *FXN* gene), long-range PCR was performed with two different primer pairs flanking the GAA repeat in intron 1 of the *FXN* gene (Fig. 1A; see methods) [2, 4]. The goal was to identify individuals who have a ‘single-band’ pattern (e.g. patients #3 and #4 in Fig. 1B), suggesting that they are homozygous for expanded alleles of the same size, versus a ‘double-band’ pattern (e.g. patients #1 and #2; Fig. 1B), indicating homozygosity for expanded alleles of different sizes. Two different primer pairs were used in order to rule out potential allele dropouts due to primer site polymorphisms. Serial dilutions were run on higher resolution gels to confirm the single-band pattern (Supplementary Fig. 1A), as opposed to unresolved alleles that were very similar in size (Supplementary Fig. 1B) or unresolvable smears due to somatic instability (Supplementary Fig. 1C). Twenty-nine unrelated individuals were identified with a defined single-band pattern (26%; Fig. 1C; 18 of whom are shown in Fig. 1D and E). Thus, approximately a quarter of FRDA patients were classifiable as being homozygous for identically-sized GAA repeats. A fresh blood sample was obtained from 25 of these 29 individuals for extraction of ultra-high molecular weight DNA suitable for longread genome sequencing.

### Identification of a recurrent proximal *FXN* gene deletion via *Alu*-mediated non-homologous recombination

**Figure 2:**
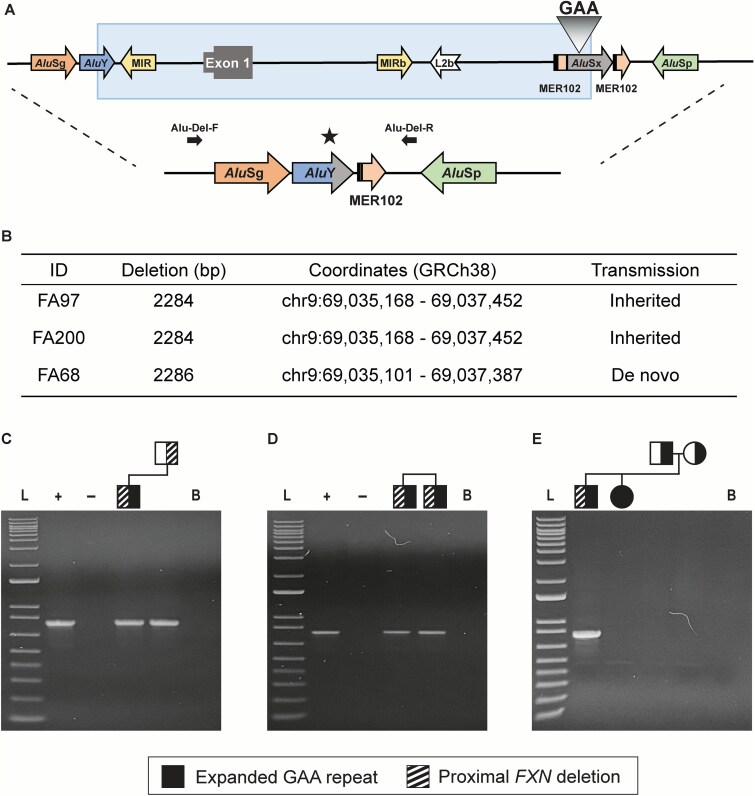
Recurrent proximal *FXN* gene deletion via *Alu*-mediated non-homologous recombination. (**A**) Schematic representation of the proximal portion of the *FXN* locus showing exon 1, the GAA repeat in intron 1, and related repetitive elements. A ~ 2.3 kb deletion (blue box) removes part of the *FXN* promoter, exon 1, 5′ portion of intron 1, including the GAA repeat (which maps at the center of an *Alu*Sx element). The deletion occurs between the 3′ half of an *Alu*Y element located upstream of exon 1 and the 3′ half of the *Alu*Sx element containing the GAA repeat, which forms a new *Alu*Y element (star). Primers used to confirm the deletion breakpoint (Alu-Del-F and Alu-Del-R) are indicated. (**B**) Summary of the proximal *FXN* deletion seen in three unrelated FRDA patients. Deletion size, genomic coordinates, and transmission patterns are indicated. (**C-E**) segregation analysis of the proximal *FXN* deletion in relatives of the three patients: FA97 (C), FA200 (D), and FA68 (E). Pedigree symbols are indicated above each gel (half-black = heterozygous for expanded GAA repeat; half-white = heterozygous for non-expanded GAA repeat; half-striped = heterozygous for proximal *FXN* deletion). L = 1 kb ladder; B = blank (no template) control; ‘+’ = positive control; ‘-’ = negative control.

Longread genome sequencing (Oxford nanopore; PromethION) revealed that three of the 25 unrelated single-band patients (12%) were compound heterozygous for a proximal *FXN* gene deletion and an expanded pure GAA repeat allele (Supplementary Fig. 2A–D; Supplementary Data 1). All three unrelated patients showed deletions of the same genomic region (~2.3 kb), which extended from the right (3′) arm of an *Alu*Y element mapping upstream of the *FXN* gene promoter to the right (3′) arm of the *Alu*Sx element within which the FRDA-related GAA repeat is located (Fig. 2A). These deletions are therefore consistent with an *Alu*-mediated, non-homologous recombination event (Supplementary Figs 3 and 4) [5]. As a result, the *FXN* gene promoter, exon 1, proximal portions of intron 1 and the GAA repeat were deleted, and a new *Alu*Y element was created (Fig. 2A). Patients FA97 and FA200 had the same deletion, which was slightly different from the deletion seen in patient FA68 (Fig. 2B), indicating at least two separate origins. These deletions resulted in removal of the upstream primer binding sites of both long-range PCR protocols employed (Fig. 1A, D and E), explaining the single-band pattern seen in these three patients.

A PCR assay was designed to detect this proximal *FXN* deletion (Fig. 2A), which confirmed the deletion in all three patients (Fig. 2C–E). Sequencing of the PCR products confirmed the same breakpoints previously detected by longread genome sequencing (Fig. 2B). Patient FA97 inherited the deletion from his father, who was himself a heterozygous carrier for this deletion (Fig. 2C). Patient FA200 shared the same deletion with a sib (Fig. 2D), suggesting that it was inherited from a carrier parent. However, FA68’s deletion, with slightly different breakpoints from FA97 and FA200, occurred as a *de novo* event (Fig. 2E). Both parents of FA68 are heterozygous for the expanded GAA repeat and do not have the proximal *FXN* deletion (Fig. 2E). FA68’s sib, FA69, who has a significantly milder phenotype (Supplementary Table 1), developed FRDA in the conventional manner, i.e. by inheriting expanded alleles from each parent. Thus, the two sibs in this nuclear family have varied molecular events leading to their FRDA phenotype. All patients with this common proximal *FXN* gene deletion (three unrelated, plus one sib) have a similarly severe FRDA phenotype (Supplementary Table 1).

### Identification of pathogenic expanded GAA-GGA composite alleles in FRDA via longread genome sequencing

**Figure 3:**
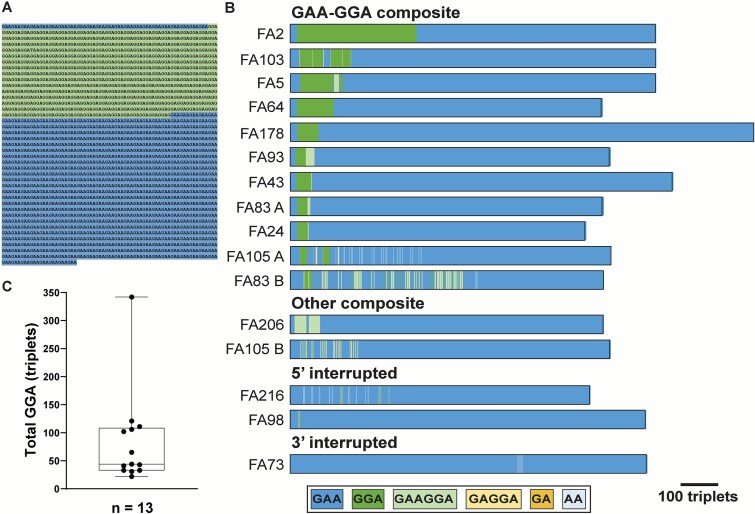
Pathogenic expanded composite alleles in FRDA. (**A**) Representative genomic sequence of an expanded composite GAA-GGA repeat. Blue indicates canonical GAA triplets; green indicates GGA triplets. (**B**) Schematic representation of expanded GAA-GGA composite alleles, other composite alleles, and alleles with 5′ and 3′ interruptions. Color-codes denote the type of non-GAA sequence, which shows a preponderance of GGA triplets (and GGA-containing hexa/pentanucleotides), that are mostly located close to the 5′ end of the expanded allele. (**C**) Box-and-whisker plot of total GGA content (in triplets) in the 13 composite alleles identified (box = interquartile range; whiskers = min and max value).

Longread genome sequencing of the remaining 22 single-band patients showed no *FXN* gene rearrangements, and all 44 *FXN* alleles were represented in the sequencing dataset. However, 11 patients showed expanded GAA alleles with substantial non-GAA content. Variable lengths of GGA tracks were a prominent feature of a majority of these alleles (Fig. 3A and B; Supplementary Data 2), thus defining a subclass of pathogenic expanded GAA-GGA composite alleles in FRDA. The GGA sequence mapped close to the 5′ end of the expanded GAA repeat (Fig. 3B). In some instances, the GGA content was contained within (GAAGGA)_n_ hexanucleotides and (GAGGA)_n_ pentanucleotides (named as ‘other composite’ alleles; Fig. 3B). Both types of composite alleles contained substantial GGA content (range: 22–342 triplets; median = 44 triplets; Fig. 3C). A few alleles showed minor non-triplet G/A interruptions in an otherwise pure GAA repeat, located either close to the 5′ or 3′ end (Fig. 3B). In every case, the expanded repeat maintained the polypurine•polypyrimidine sequence composition. Notably, 28 of the 44 alleles (64%) were pure GAA repeats, hence most people with composite alleles were compound heterozygous for a pure GAA allele and a composite allele (Supplementary Fig. 5). The length of expanded composite/interrupted alleles was not significantly different from expanded pure GAA repeats.

### Detection of pathogenic expanded GAA-GGA composite alleles in FRDA via modified PCR conditions

**Figure 4:**
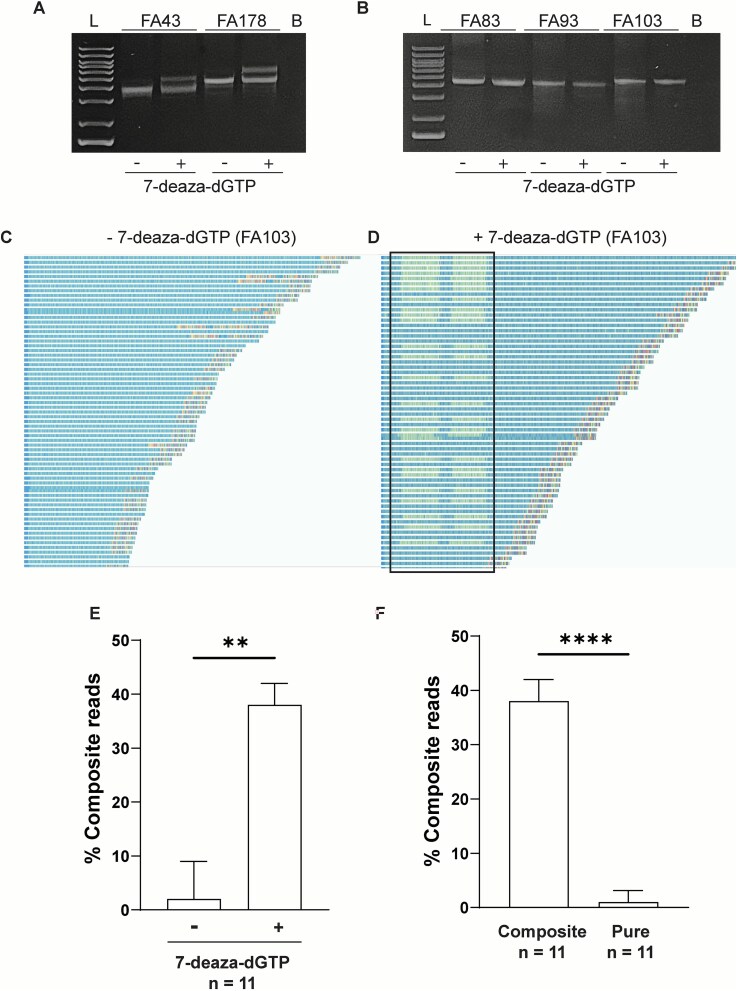
Detection of pathogenic expanded composite alleles using modified PCR conditions. (**A**) Long-range PCR with 7-deaza-dGTP in FRDA patients known to have the expanded composite allele via longread genome sequencing reveals the presence of a composite allele. Note conversion of the ‘single-band’ pattern in two such patients (minus 7-deaza-dGTP) into a ‘double-band’ pattern (plus 7-deaza-dGTP). L = ladder; B = blank (no template) control. (**B**) the expanded composite allele is not always revealed via addition of 7-deaza-dGTP (three patients known to have expanded composite alleles via longread genome sequencing are shown). L = ladder; B = blank (no template) control. In such patients, the expanded composite allele is similar in length to the expanded pure allele. (**C, D**) IGV pileups of longread deep sequencing reads from purified amplicons generated without (C) and with (D) 7-deaza-dGTP for patient FA103 reveal the presence of the composite allele only in the presence of 7-deaza-dGTP. Blue = GAA triplets; green = GGA triplets. The fixed left-edge is the start of the GAA repeat and the variable right-edge is the site of the transposase entry into the expanded repeat (the multicolor pattern represents the adapter sequence). (**E**) Composite reads (%) detected via longread deep sequencing of amplicons in 11 patients known to have expanded composite alleles, minus and plus 7-deaza-dGTP. ^**^ = *p* < 0.001 (Mann–Whitney test). (**F**) Composite reads (%) detected via longread deep sequencing of amplicons generated in the presence of 7-deaza-dGTP in patients known to have expanded composite alleles (n = 11) versus those known to have only expanded pure GAA alleles (n = 11). ^****^ = *p* < 0.0001 (Mann–Whitney test).

The visible allele via long-range PCR did not always match the length of the composite allele detected via longread genome sequencing, indicating that composite alleles were not being amplified efficiently. A modified PCR protocol, primarily the addition of 7-deaza-dGTP (see Methods) [6, 7], remedied this situation, suggesting that the relatively high G/C content of the GGA motif (versus GAA) contributed to the difficulty in amplification via conventional PCR. Thus, when the expanded GAA-GGA composite allele was different in size from the pure GAA allele, the single-band changed to a double-band pattern (Fig. 4A). However, given that composite and pure alleles are quite often the same length, a distinct double-band pattern was not always seen in patients known to have composite alleles (Fig. 4B). In order to visualize the composite allele, the PCR amplicons generated with/without 7-deaza-dGTP were subjected to longread sequencing (Oxford nanopore; PromethION). This clearly showed that addition of 7-deaza-dGTP was required to detect the composite allele. For instance, patient FA103, who remained with a single-band pattern upon PCR with 7-deaza-dGTP (Fig. 4B), showed composite alleles via deep sequencing of amplicons generated with 7-deaza-dGTP, but not in its absence (Fig. 4C versus Fig. 4D). Indeed, in our series of patients with the single-band pattern and known to have a composite allele, addition of 7-deaza-dGTP permitted efficient detection of the composite allele, which accounted for ~ 40% of the reads via deep sequencing (Fig. 4E). The efficiency of 7-deaza-dGTP to detect the expanded composite allele was dependent on the total GGA content and the length of the longest GGA track within the composite allele (Supplementary Fig. 6A and B). This latter dependence was not seen for total GAA content or the total length of the expanded allele (Supplementary Fig. 6C and D). Some composite alleles were detectable at low levels in the absence of 7-deaza-dGTP (Supplementary Fig. 7), but it was required for the efficient detection of most composite alleles (Fig. 4E). The Oxford nanopore platform is known to generate errors during sequencing, but this was found to be quite low. Deep sequencing of the amplicons generated in the presence of 7-deaza-dGTP detected composite alleles in patients known to have composite alleles by longread genome sequencing but not in individuals known to have only pure GAA alleles (Fig. 4F).

A validation cohort of 14 additional FRDA patients with the single-band pattern was analyzed by PCR with and without 7-deaza-dGTP followed by longread deep sequencing of the amplicons. Similar to the larger cohort, this validation cohort revealed seven new composite alleles, six of which were the common expanded GAA-GGA composite allele (Supplementary Fig. 8; Supplementary Data 3), which confirmed that a substantial number of single-band FRDA patients have composite alleles.

### False-negative outcomes in heterozygous carriers of expanded GAA-GGA composite alleles

**Figure 5:**
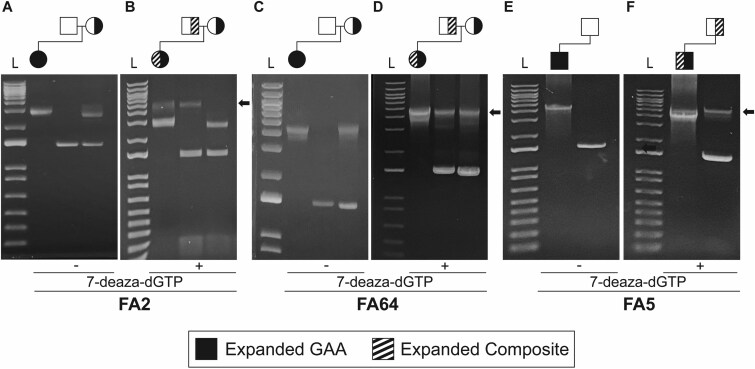
False-negative outcomes in heterozygous carriers of expanded composite alleles. (**A, C, E**) conventional long-range PCR analysis in parents of patients with expanded composite alleles showed that one parent (the father in all three cases) is not a heterozygous carrier. (**B, D, F**) long-range PCR in the presence of 7-deaza-dGTP, in contrast, reveals the composite allele in the parent who previously didn’t seem to be a heterozygous carrier (black arrows). Pedigree symbols are indicated above each gel (half-black = heterozygous for expanded GAA repeat; half-striped = heterozygous for expanded composite allele; unfilled = normal allele plus absence of expanded allele). L = 1 kb ladder.

First-degree relatives of FRDA patients are often tested for carrier status determination. Since parents are almost always heterozygous carriers, we tested the parents of three patients known to be compound heterozygous for an expanded GAA-GGA composite allele and a pure GAA allele. In each case, the standard long-range PCR assay (i.e. minus 7-deaza-dGTP) showed a single band pattern in the proband, and that one of the parents was not a heterozygous carrier (Fig. 5A, C, E). This is consistent with the previous observation that composite alleles are resistant to amplification via the conventional PCR-based assay. The modified long-range PCR (i.e. plus 7-deaza-dGTP) revealed that these parents were indeed heterozygous carriers of an expanded allele (Fig. 5B, D, F), and deep sequencing of the amplicons confirmed that they carried the expanded GAA-GGA composite allele.

### Detection of pathogenic composite and interrupted alleles in ‘double-band’ FRDA patients

**Figure 6:**
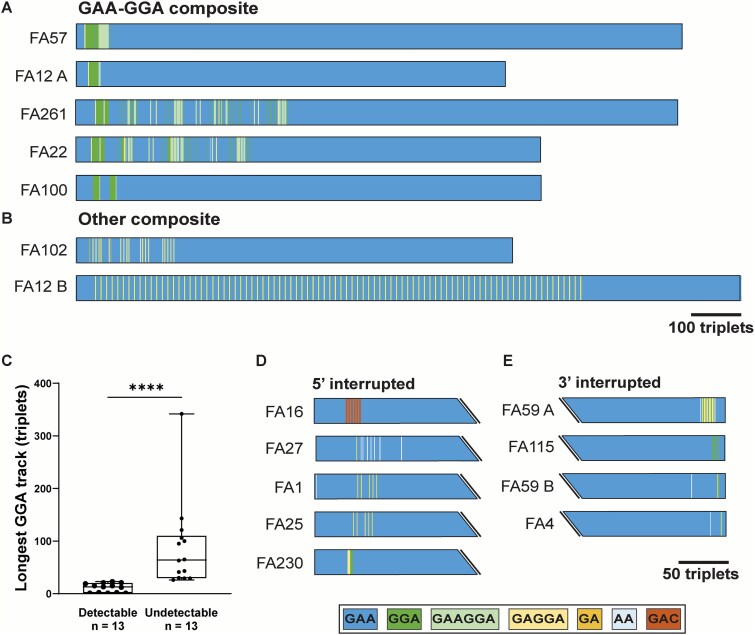
Pathogenic composite and interrupted alleles in ‘double-band’ FRDA patients. (**A, B**) schematic representation of expanded GAA-GGA composite alleles, and other composite alleles. (**C**) Box-and-whisker plot of longest GGA track (in triplets) in the 13 composite alleles detectable by conventional long-range PCR versus 13 composite alleles that were not detectable (i.e. in FRDA with the ‘single-band’ pattern). Box = interquartile range; whiskers = min and max value. ^****^ = *p* < 0.0001 (Mann–Whitney test). (**D, E**) schematic representation of expanded alleles with 5′ and 3′ interruptions. Color-codes denote the type of non-GAA sequence.

In order to determine the prevalence of composite alleles in FRDA we additionally tested a cohort of 59 FRDA patients who had the ‘double-band’ pattern on conventional testing, i.e. homozygous for expanded GAA repeats of different sizes. We reasoned that composite alleles with low GGA content and other types of smaller sequence interruptions may be present in such patients. Long-range PCR plus 7-deaza-dGTP followed by deep sequencing of the amplicons on the Oxford nanopore platform was performed. Of the 118 alleles, 102 (86%) were pure GAA repeats, indicating that a majority of double-band FRDA patients do not have composite/interrupted alleles. However, we did detect a total of 7 composite alleles, 5 of which were expanded GAA-GGA composite alleles (Fig. 6A and B; Supplementary Data 4). The two ‘other composite’ alleles featured multiple instances of (GAGGA)_n_ pentanucleotides, with one of them (FA12B) having 88 repeats of [(GAGGA)_1_(GGA)_1_(GAA)_8_]. As expected, the longest GGA track in these readily amplifiable alleles (hence ‘double-band’ pattern) was significantly shorter than in the non-amplifiable composite alleles seen in single-band patients (Fig. 6C). A variety of 5′ and 3′ interruptions were also detected (Fig. 6D and E). Interestingly, one of the 5′ interrupted alleles (in FA16) contained multiple units of (GA**C**GA**C**GAA), different from the pathognomonic polypurine•polypyrimidine sequence composition seen in FRDA-associated expanded alleles.

## Discussion

Longread sequencing revealed a previously unrealized level of heterogeneity among pathogenic alleles in FRDA. It is estimated that ~ 20% of patients are heterozygous for an expanded composite allele, i.e. compound heterozygous for an expanded composite repeat and a pure GAA repeat. Most of these individuals are currently diagnosed as being homozygous for expanded alleles of the same size, because the G/C-rich composite allele is commonly missed by conventional PCR-based assays. Indeed, as many as 40% of FRDA patients who are said to be homozygous for expanded alleles of the same size will have one pathogenic expanded GAA-GGA composite allele. Minor sequence interruptions at the 5′ and 3′ end of expanded GAA repeat have been known to occur in FRDA [8, 9], and these were also observed in ~ 10% of the present series of patients. However, it is now clear that major non-GAA interruptions occurring near the 5′ end of pathogenic repeats are quite common in FRDA. Variable lengths of tandem GGA triplets constitute a very common non-GAA motif, and is the defining feature of the pathogenic expanded GAA-GGA composite allele.

This new category of allelic variability provides additional opportunities for the refinement of genotype–phenotype correlation in FRDA, which has thus far relied primarily on the variable length of the expanded repeat [3, 4, 10–13]. Indeed, these composite alleles have been missing from the genotypes considered for phenotypic correlations, even in studies specifically designed to evaluate the effect of non-GAA interruptions [8, 9]. In addition, patients with an inordinately severe FRDA phenotype that also appear to be homozygous for expanded alleles of the same size should be considered as candidates for the recurrent proximal *FXN* gene deletion.

It has been suggested that major non-GAA interruptions in typical FRDA-related expanded alleles are rare [14]. When detected, they have tended to be associated with relatively short expanded alleles (<300 triplets), which either produced a very late onset FRDA phenotype [15], or were non-pathogenic [16, 17]. This type of interrupted and relatively short expanded allele is known to have very low levels of somatic instability [18]. Indeed, base-editing strategies designed to incorporate non-GAA sequence interruptions within the expanded GAA repeat is being considered as a therapeutic strategy to reduce somatic repeat expansions *in vivo* [19]. However, our findings show that large expanded alleles with substantial non-GAA interruptions are pathogenic and are quite prevalent in Friedreich ataxia.

The observation of pathogenic expanded composite alleles (and those with other types of interruptions) in FRDA contrasts with SCA27B, the other genetically-determined ataxic phenotype associated with an expanded GAA repeat (located in intron 1 of the *FGF14* gene) [20]. Pathogenic alleles causing SCA27B are typically pure GAA tracks of > 300 triplets (alleles with 250–299 GAA triplets are associated with reduced penetrance). Non-pathogenic *FGF14* alleles containing the equivalent of > 250 (or even > 300) triplets are typically interrupted with non-GAA sequences [21, 22]. It is unclear why repeat interruptions at the *FXN* locus allow for disease pathogenesis, but those at the *FGF14* locus do not (despite both repeats mapping to intron 1 of their respective transcriptional units). Possible explanations include the following: (i) FRDA-related pathogenic composite alleles are considerably longer and have substantial tracks of pure GAA sequence compared with non-pathogenic alleles at the *FGF14* locus; (ii) non-pathogenic interrupted alleles with > 250 triplets at the *FGF14* locus have relatively short pure GAA tracks [23], which is similar to the interrupted GAA alleles at the *FXN* locus that are either non-pathogenic [16, 17], or associated with a very late onset of disease [15]; and (iii) the pathogenic consequences of the expanded GAA repeat may be different at the *FXN* and *FGF14* loci; for instance, in the *FXN* transcriptional unit the repeat track is in the (GAA•TTC)_n_ orientation, which is known to be more transcriptionally repressive compared with the (TTC•GAA)_n_ orientation [24], which is seen at the *FGF14* locus [23].

The inclusion criterion for this study was that patients must have had a genetic test (from a commercial testing lab) and have a molecular diagnosis of FRDA due to homozygosity for the expanded GAA repeat (thus, base substitutions, small indels, etc., were not studied here). All patients were subsequently retested in our research lab, which showed a reasonable level of congruence in the estimation of repeat lengths. There were occasional patients for whom the commercial lab reported expanded alleles of the same size, which we resolved into two distinct bands. However, each of the 29 patients in the prospective series of 112 whom we determined to have the ‘single-band’ pattern, were similarly determined by the commercial lab to be homozygous for identically-sized expanded GAA repeats. As seen here, many of these individuals have expanded composite alleles (and a few of them have *FXN* gene deletions) that were missed by long-range PCR. This indicates that commercial gene testing presently misses composite alleles (and the proximal *FXN* gene deletion). This is not necessarily a major clinical problem, because the molecular diagnosis of FRDA is still appropriately made. However, false-negative results in carrier status determination among relatives of these patients is unfortunately a distinct possibility. Another PCR-based protocol often used to diagnose FRDA, repeat-primed PCR [25], would likely also miss detecting the G/C-rich composite alleles. Similar to long-range PCR, detection of the amplifiable pure GAA allele paired with the absence of a normal allele will still result in the accurate diagnosis of FRDA. However, carrier status determination for heterozygous carriers of the composite allele would likely be fraught. *Short-read* whole genome sequencing followed by use of algorithms such as ExpansionHunter/Dragen, should be able to accurately identify the presence of expanded composite alleles in FRDA patients and in heterozygous carriers [26], but may not be able to characterize the precise sequence content of such alleles. Commercial testing labs, and research labs that require accurate determination of the size of both expanded alleles, should consider updating their detection protocol, either by using the PCR conditions we describe here or via other methods to adequately address the relatively high G/C content of composite alleles. Furthermore, whereas the expense and logistics of longread *genome* sequencing are presently prohibitive for routine diagnostic testing, the detailed characterization of expanded composite alleles via longread sequencing of the repeat-containing *amplicons* generated in the presence of 7-deaza-dGTP is a more manageable strategy.

## Materials and methods

### Study participants

Peripheral blood samples were collected in purple-top/EDTA tubes from a prospective series of individuals with a confirmed molecular diagnosis of FRDA at the Children’s Hospital of Philadelphia (CHOP). Only individuals who were homozygous for the expanded GAA repeat in the *FXN* gene, as determined via commercial DNA testing, were included in this study. Fresh blood samples were shipped overnight on ice via courier service for processing and analysis at the University of Oklahoma Health Sciences Center (OUHSC) in Oklahoma City. Research protocols were approved by the institutional review boards at both institutions (CHOP IRB 25–023116 and OUHSC IRB 18177).

### DNA isolation

Genomic DNA used for PCR was extracted using the QIAamp DNA Blood Midi Kit (Qiagen; 51 185). Ultra-high-molecular weight DNA used for longread genome sequencing was extracted from whole blood using the Nanobind CBB kit (PacBio; 102–207–600). Long-range PCR amplicons were purified for longread sequencing using the Wizard SV Gel and PCR Clean-Up System (Promega; A9282).

### Detection of the proximal *FXN* deletion

The recurrent proximal *FXN* deletion was detected by PCR using primers: Alu-Del-F: 5′-ACTTTCACAATTTGCATCCCTTT-3′ and Alu-Del-R: 5′-CAGGGGTGGAAGCCCAATAC-3′. PCR cycling conditions were as follows: initial denaturation at 94°C for 3 minutes followed by 30 cycles of 94°C for 1 minute, annealing at 58°C for 1 minute, and extension at 72°C for 1 minute, followed by a final extension at 72°C for 10 minutes.

### Long-range PCR of pure and composite expanded alleles

GAA repeat lengths were determined by long-range PCR using the AccuStart Long Range SuperMix (QuantaBio; 95 199–100). Duplicate analysis was performed via generation of two different amplicons, in order to determine the presence of a “double-band” or “single-band” pattern, for each individual. The two different primer sets that flank the GAA repeat in intron 1 are as follows: (a) GAA-104F & GAA-629R, as described previously [4] (which generates a product of 499 bp + repeats); (b) T3F: 5’-GCGCGCAATTAACCCTCACTAAAGGGAACA*GGAGGGATCCGTCTGGGCAAAGG*-3′ & T7R: 5’-CGCGCGTAATACGACTCACTATAGGGCGA*CAATCCAGGACAGTCAGGGCTTT*-3′ (which generates a product of 1429 bp + repeats). The italicized portion of T3F and T7R match the sequence of primers Bam and 2500F, which were used in the original description of the expanded GAA repeat in FRDA [2]. Primers T3F and T7R primers were generated for another project, and in our experience, they perform more consistently compared with the original Bam and 2500F primers for the detection of expanded repeats. The following PCR cycling conditions were used for GAA-104F and GAA-629R: initial denaturation at 93°C for 3 minutes followed by 10 cycles of 93°C for 15 s, annealing at 65°C for 30 s, and extension at 68°C for 3 minutes, followed by 25 cycles of 93°C for 15 s, annealing at 65°C for 30 s, and extension at 68°C for 3 minutes, with a 15 s increment in extension period per cycle, followed by a final extension at 68°C for 7 minutes. The following PCR cycling conditions were used for T3F and T7R: initial denaturation at 93°C for 3 minutes followed by 20 cycles of 93°C for 15 s, and annealing plus extension at 68°C for 5 minutes, followed by 17 cycles of 93°C for 15 s, and annealing plus extension at 68°C for 5 minutes, with a 15 s increment in extension period per cycle, followed by a final extension at 68°C for 7 minutes. For amplification of composite alleles, the same long-range PCR conditions with T3F and T7R primers were used, with the addition of 0.2 mM 7-deaza-dGTP (New England Biolabs; N0445L) and 0.1 M betaine (Millipore Sigma; B0300). Detection of composite alleles was performed as two separate reactions; i.e. plus and minus 7-deaza-dGTP and betaine.

### Detection of composite alleles by longread genome sequencing

Whole genome shotgun (WGS) libraries were prepared using the Ligation Sequencing Kit V14 (Oxford Nanopore Technologies; SQK-LSK114) as per the manufacturer’s protocol. Briefly, DNA samples underwent nick repair and end-prep followed by ligation with ONT-specific sequencing adapters. These libraries were loaded onto a R10.4.1 PromethION flowcell (FLO-PRO114M) and run on the ONT PromethION 2 Solo instrument. On-instrument basecalling was set to Fast Accuracy model, 400 bps, using MinKNOW v23.07.12 and Guppy v7.1.4. Whole genome sequencing was performed for most samples. However, a few samples were analyzed by adaptive sampling in order to sequence only the genomic interval spanning the *FXN* locus (chr9:69007181-69 236 526 in GRCh38). Adaptive sampling was performed during the sequencing run, set to Enrich Reference. The reference was generated as a custom track for the *FXN* locus in the UCSC Genome Browser using the View > DNA Sequence tool with Mask Repeats set to ‘N.’ Dorado v0.8.0 with default parameters was used for re-basecalling and alignment to the human reference genome (GRCh38). Sequence reads were filtered to retain only those overlapping the *FXN* locus (chr9:69035752–69 079 076 in GRCh38) using SAMtools v1.21. Mapping statistics and depth of coverage were computed using the Epi2Me Labs alignment workflow, which incorporates Minimap2, Bamstats, and Mosdepth. The resulting aligned and sorted BAM files were analyzed using the Epi2Me Labs human variation workflow to detect phased small variants (via Clair3 and SnpEff), structural variants (via Sniffles2), copy number variants (via Spectre), and short tandem repeats (via Straglr). Together, these workflows were used to characterize the *FXN* locus for substitutions, insertions/deletions, and copy number variants. The short tandem repeat workflow, along with analysis of individual aligned reads, was used to characterize the *FXN* GAA repeat region and to identify composite and interrupted alleles.

### Detection of composite alleles by longread sequencing of long-range PCR amplicons

Long-range PCR (with T3F and T7R primers), plus and minus 7-deaza-dGTP and betaine, was performed for each individual. Both purified amplicons were analyzed via longread deep sequencing. Amplicon libraries were prepared using Rapid Sequencing Kit V14 (Oxford Nanopore Technologies; SQK-RAD114) as per the manufacturer’s protocol. Briefly, DNA from each sample was tagmented with ONT-specific transposase adapters followed by attachment of sequencing adapters via click chemistry. Libraries were loaded onto a R10.4.1 PromethION flowcell (FLO-PRO114M) and run on the ONT PromethION 2 Solo instrument. Basecalling was set to High Accuracy, 400 bps, using MinKNOW v23.07.12 and Guppy v7.1.4. Reads were re-basecalled and aligned to the human reference genome (GRCh38) using Dorado v0.8.0 with default parameters. Reads overlapping the 5′ or 3′ ends of the GAA region in intron 1 of the *FXN* gene were extracted and visualized in the Integrative Genomics Viewer (IGV). Composite allele sequence frequency was determined by randomly selecting 100 reads overlapping the 5′ and 3′ end of the GAA repeat. Reads were included only if they contained at least 200 bp of the GAA repeat. While both ends of the repeat sequence were analyzed, composite sequences were mostly detected at the 5′ end in our series. The number of motif-positive and motif-negative reads at each boundary was counted to estimate the percentage of composite reads and pure GAA reads, respectively.

### Determination of the consensus sequence of composite alleles

The consensus sequence for each of the composite alleles was determined using a combination of the multiple sequence reads obtained via longread genome sequencing and amplicon sequencing. The total allele length was determined based on the allele sizes calculated via gel electrophoresis. All composite alleles are graphically depicted in the associated figures (and supplemental figures), and the actual consensus sequences are provided in Supplementary Data 1–4.

### Statistical analyses

Statistical analyses were performed using GraphPad Prism v10.4.2 and Epi2Me Labs. The Mann–Whitney test was used to compare medians and interquartile ranges for non-normally distributed data. Simple linear regression and Pearson correlation was applied to assess the relationship between composite allele characteristics and PCR amplification efficiency under modified PCR conditions. Read depth and quality metrics were obtained using the Epi2Me Labs alignment workflow.

## Supplementary Material

Devore_et_al_Supplementary_data_1_Final_ddaf190

Devore_et_al_Supplementary_data_2_Final_ddaf190

Devore_et_al_Supplementary_data_3_Final_ddaf190

Devore_et_al_Supplementary_data_4_Final_ddaf190

Devore_et_al_Supplementary_Materials_HMG_final_ddaf190

## Data Availability

All genomic and PCR amplicon longread sequence files have been deposited in the National Center for Biotechnology Information (NCBI) Sequence Read Archive (SRA) under BioProject accession number PRJNA1304994. Per IRB protocol, only sequence reads overlapping the *FXN* locus (chr9:69035752–69 079 076 in GRCh38) are made publicly available. The consensus sequences determined for all composite alleles are provided in Supplementary Data files 1-4.
