# Supplementary material for: Unrecognized high prevalence of expanded composite repeats in Friedreich ataxia
Source: Hum Mol Genet. 2025 Dec 23;35(3):ddaf190. doi: 10.1093/hmg/ddaf190 (PMC13158230; doi:10.1093/hmg/ddaf190)
Supplement: Devore_et_al_Supplementary_Materials_HMG_final_ddaf190 [file devore_et_al_supplementary_materials_hmg_final_ddaf190.pdf]

Supplementary Materials

This file includes:

Supplementary Figures. 1-8

Supplementary Table 1

Description of supplementary Data 1-4

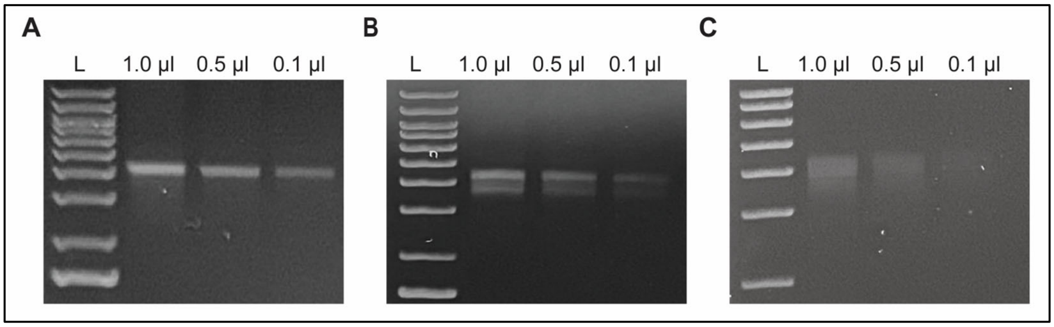

Supplementary Figure 1. Serial dilutions to confirm "single-band" pattern. Dilution series (1.0 µL, 0.5 µL, 0.1 µL) of long-range PCR product from FRDA patients showing: **(A)** single-band pattern (only this type of result prompted further analysis); **(B)** likely double-band pattern, and not considered as a definite single-band (note: the intensity of smaller products of long-range PCR are usually more intense due to PCR bias, but this is inverted here); **(C)** smeary band pattern, suggestive of somatic instability, and not considered as a definite single-band. L = 1 kb ladder.

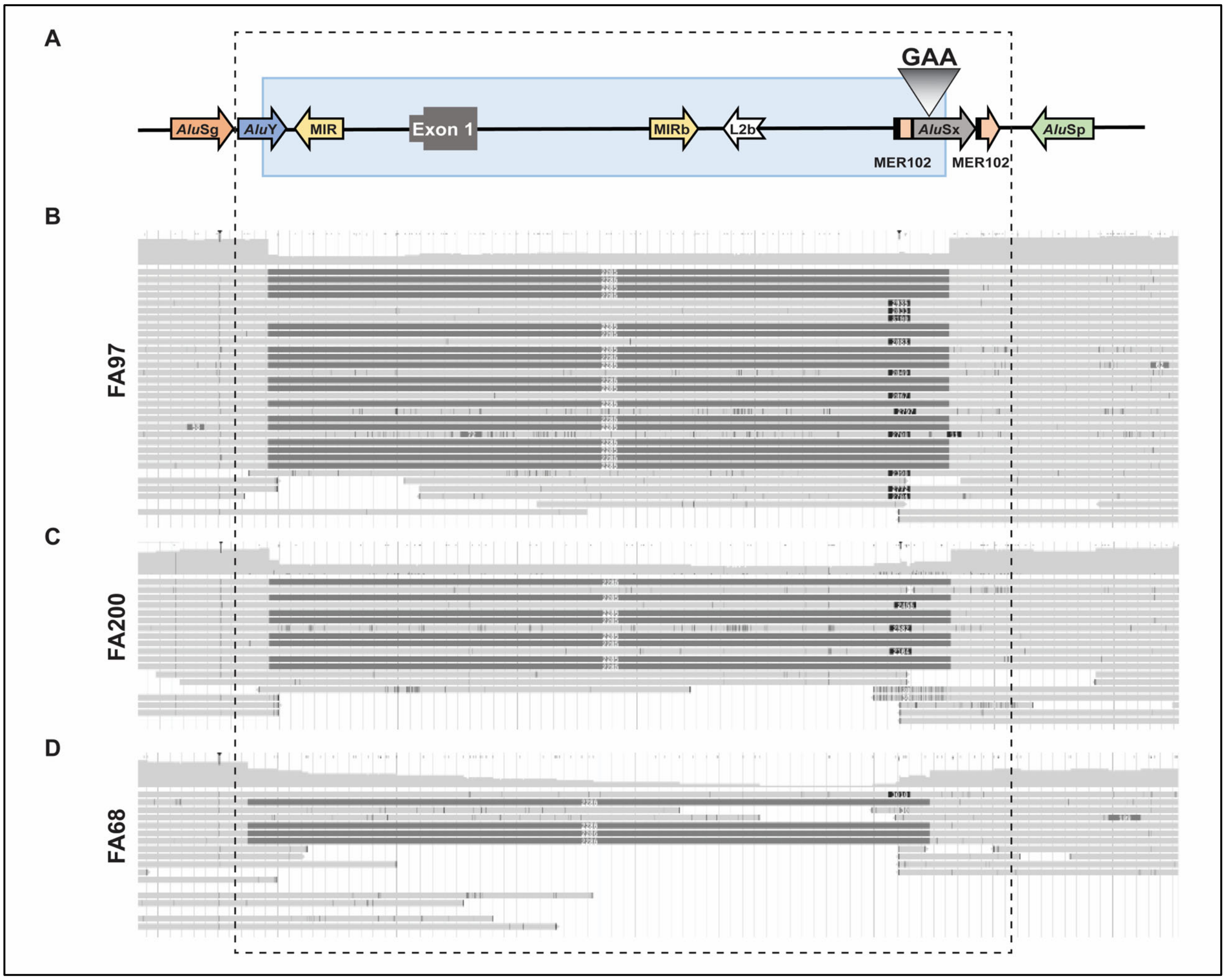

Supplementary Figure 2. Proximal *FXN* gene deletion in FRDA revealed by longread genomic sequencing. (A) Schematic representation of the proximal portion of the *FXN* locus showing exon 1, the GAA repeat in intron 1, and related repetitive elements. A ~2.3 kb deletion (blue box) removes part of the *FXN* promoter, exon 1, 5' portion of intron 1, including the GAA repeat (which maps at the center of an *Alu*Sx element). **(B–D)** Longread sequencing pileup plots showing the proximal *FXN* deletion (dark grey lines represent the missing region; light gray lines represent the non-deleted sequence) in patients FA97, FA200, and FA68. FA97 and FA200 have identical deletion breakpoints, while FA68 has slightly different breakpoints (see Fig. 2B).

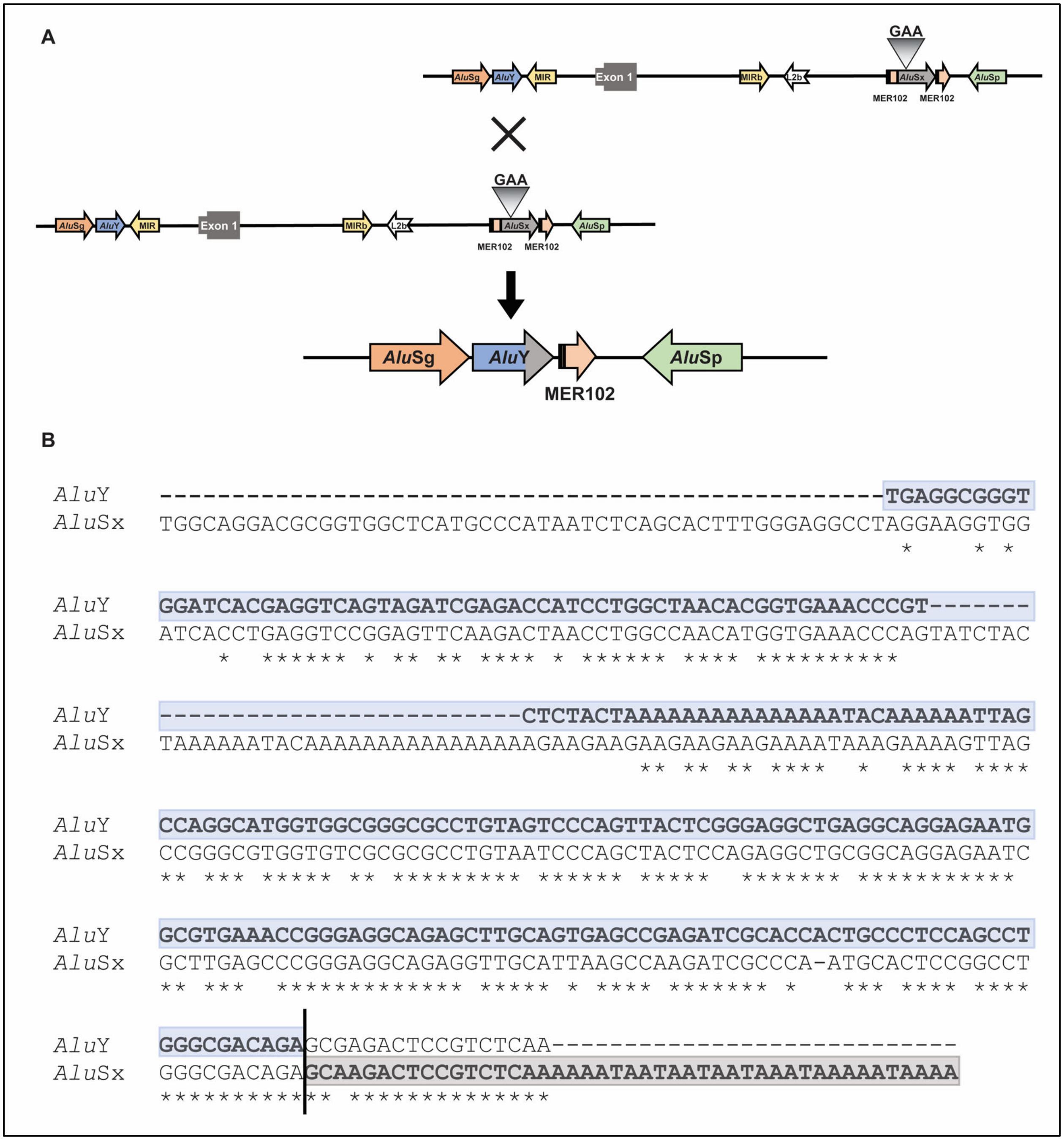

Supplementary Figure 3. Model of *Alu*-mediated non-homologous recombination resulting in the common proximal *FXN* deletion in FA97 and FA200. (A) Schematic representation of the 5′ region of the *FXN* locus, illustrating *Alu*-mediated slipped mis-pairing (between the 3′ half of an *Alu*Y element located upstream of exon 1 and the 3′ half of the *Alu*Sx element containing the GAA repeat) and non-homologous (unequal) recombination, which results in a new *Alu*Y element at the deletion junction. **(B)** Sequence alignment of the mis-paired *Alu* elements. The blue outline represents the 5′ portion of the newly formed *Alu* derived from the *Alu*Y element and the grey outline indicates the 3′ portion derived from the *Alu*Sx element. The black vertical line marks the common deletion breakpoint seen in patients FA97 and FA200 (and their relatives). Asterisks denote identical bases.

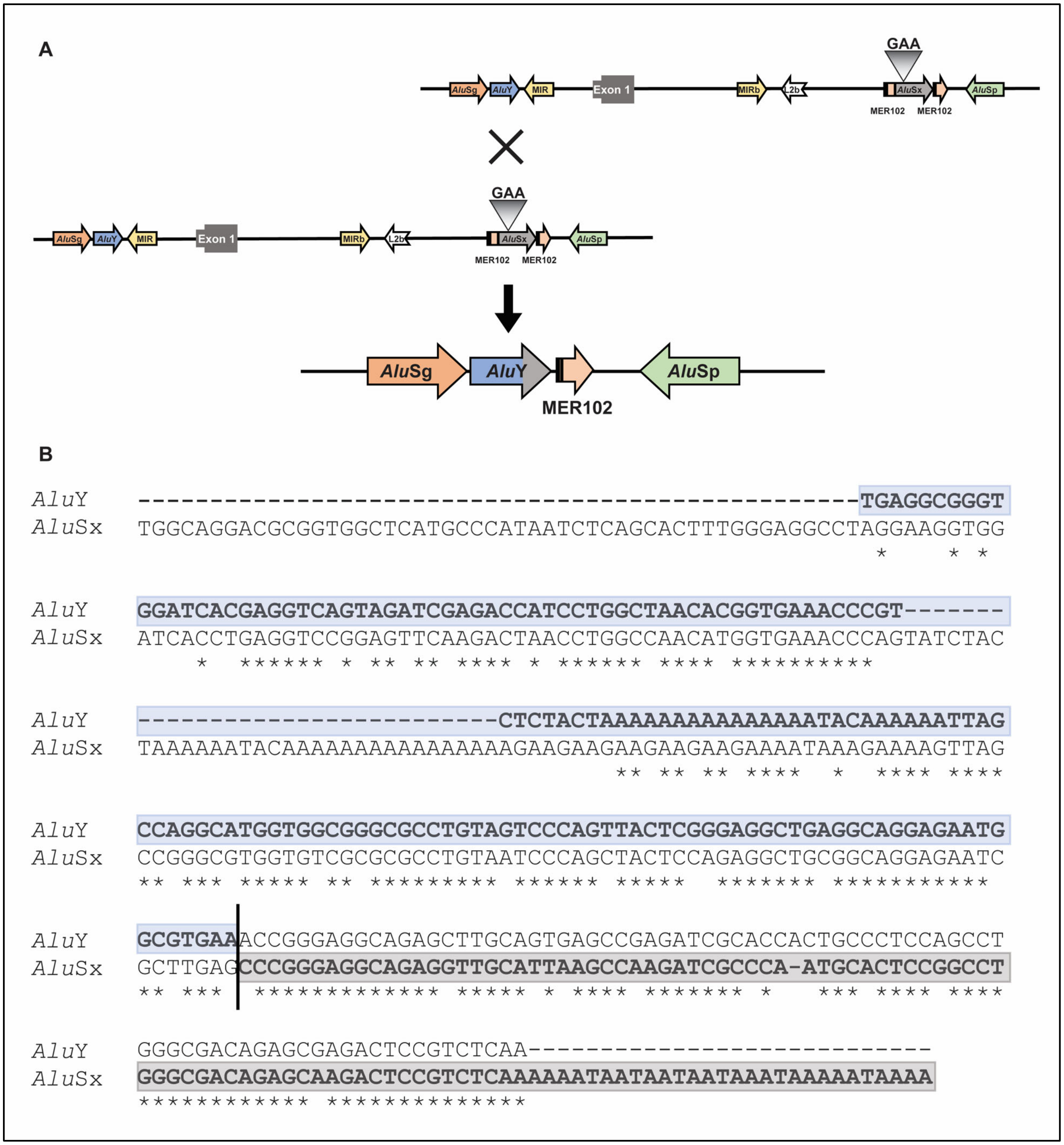

Supplementary Figure 4. Model of *Alu*-mediated non-homologous recombination resulting in the common proximal *FXN* deletion in FA68. (A) Schematic representation of the 5′ region of the *FXN* locus, illustrating *Alu*-mediated slipped mis-pairing (between the 3′ half of an *Alu*Y element located upstream of exon 1 and the 3′ half of the *Alu*Sx element containing the GAA repeat) and non-homologous (unequal) recombination, which results in a new *Alu*Y element at the deletion junction. **(B)** Sequence alignment of the mis-paired *Alu* elements. The blue outline represents the 5′ portion of the newly formed *Alu* derived from the *Alu*Y element, and the grey outline indicates the 3′ portion derived from the *Alu*Sx element. The black vertical line marks the *de novo* deletion breakpoint seen in patient FA68. Asterisks denote identical bases.

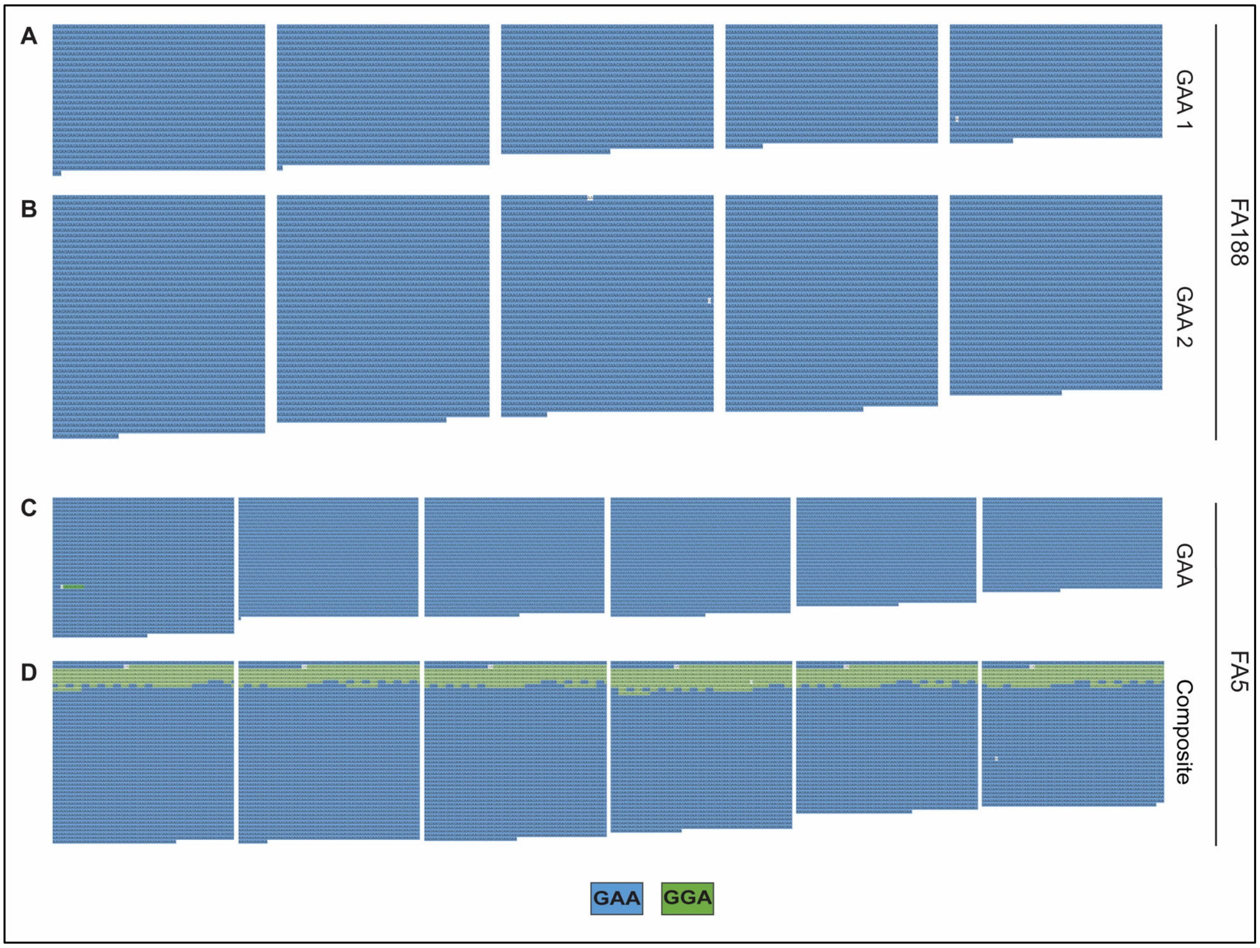

Supplementary Figure 5. Detection of expanded composite alleles by longread genomic sequencing. Representative longread sequences of the *FXN* GAA repeat using the Oxford Nanopore platform. **(A, B)** Sequence reads from a patient (FA188) with the "double-band" pattern on conventional long-range PCR showing two expanded pure GAA repeat alleles (GAA1 and GAA2). **(C, D)** Sequence reads from a patient (FA5) with the "single-band" pattern on conventional long-range PCR showing compound heterozygosity for one expanded pure GAA repeat allele and one expanded GAA-GGA composite allele. Blue segments represent GAA triplets, and green segments represent GGA triplets.

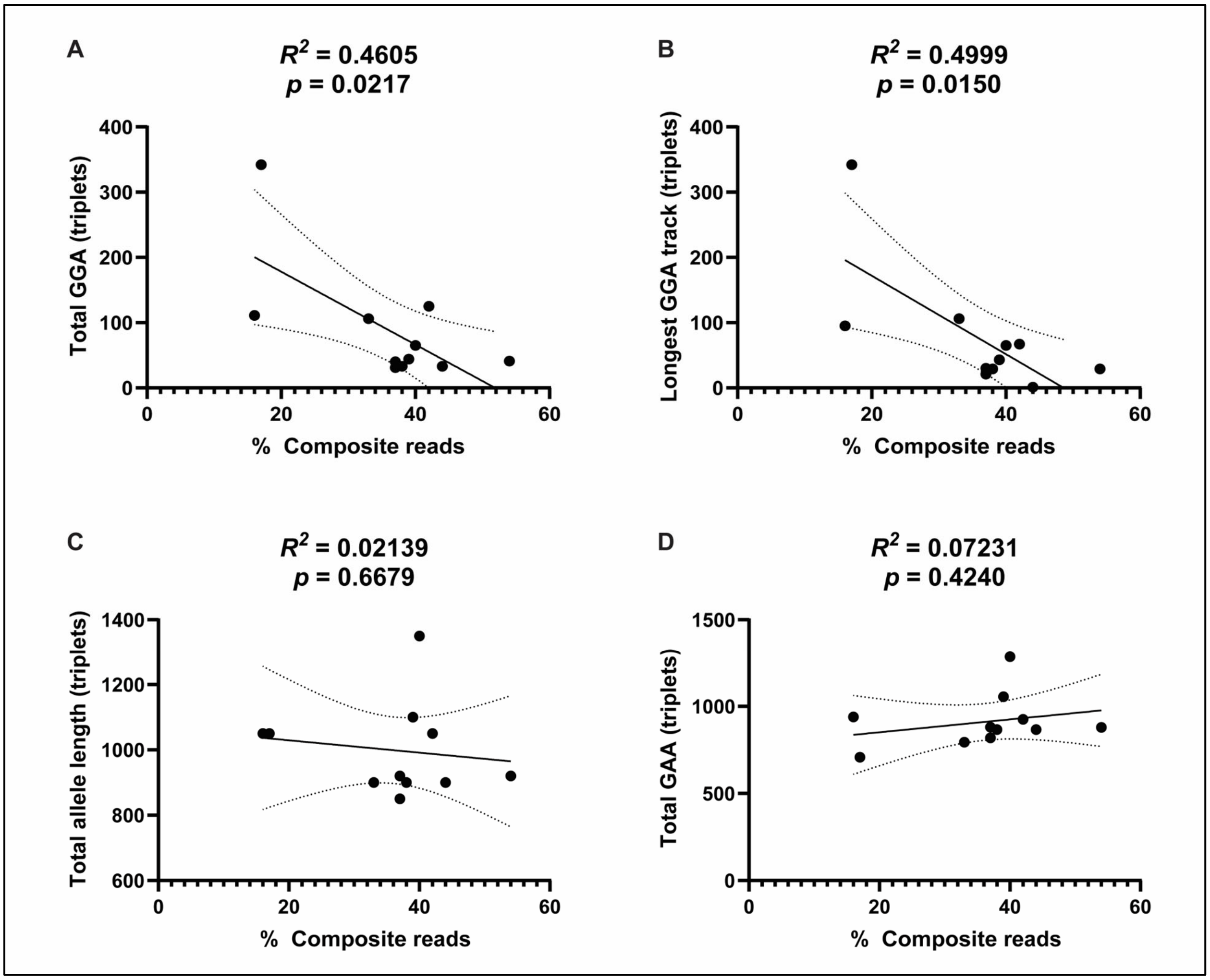

Supplementary Figure 6. Correlation of composite allele sequences and amplification efficiency in the presence of 7-deaza-dGTP. Simple linear regression of the proportion of composite reads (X-axis) detected via longread deep sequencing of long-range PCR amplicons (in the presence of 7-deaza-dGTP) versus various sequence features of the composite allele in 11 FRDA patients (Y-axis). A statistically significant positive correlation is observed for **(A-B)** total GGA content (A), and the longest GGA tract (B). **(C-D)** No such correlation was noted for **(C)** total allele length, or **(D)** total GAA content. R^2 and *p* values from Pearson correlation are noted on each plot. Solid lines represent the best-fit regression line, and dotted lines denote 95% confidence intervals.

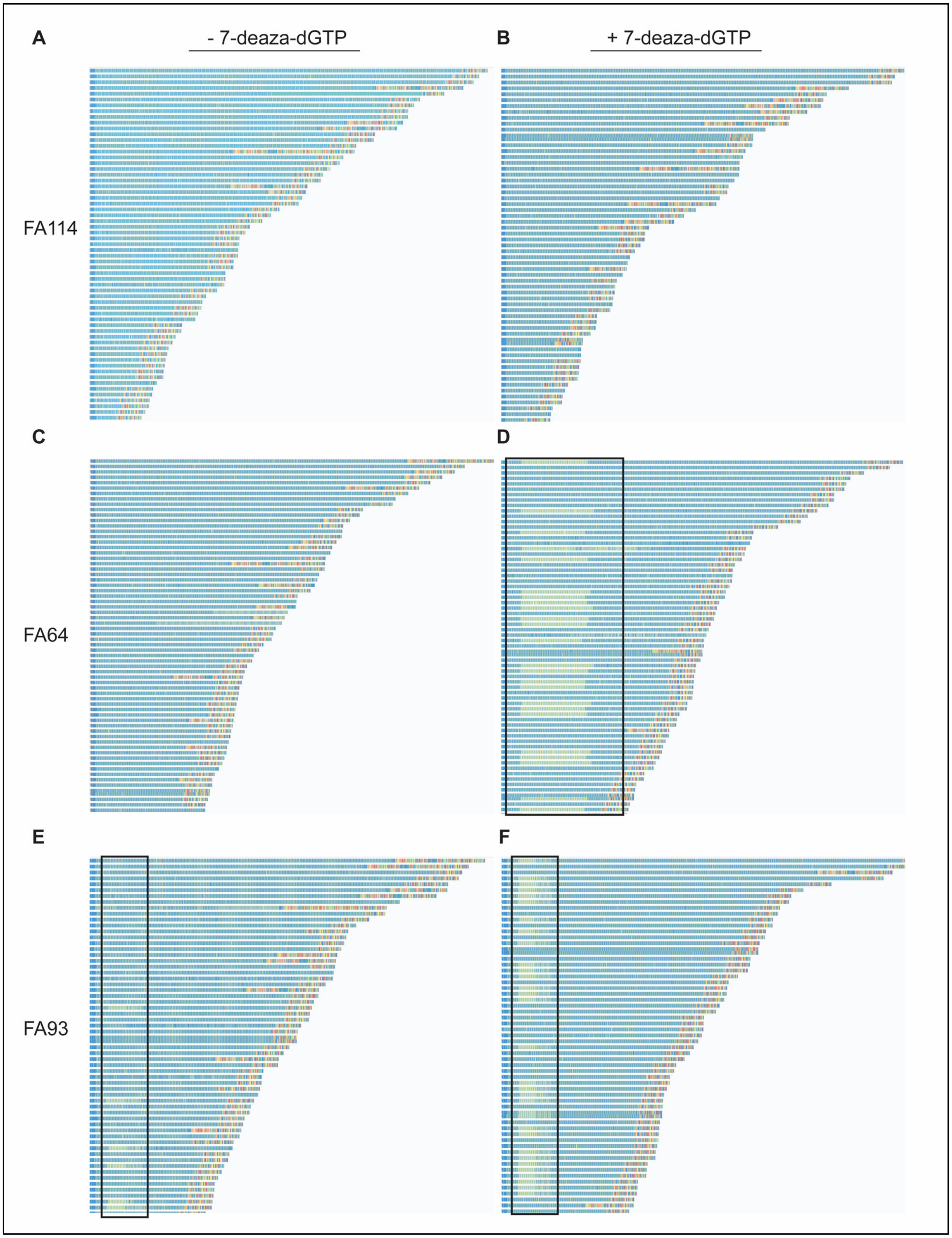

Supplementary Figure 7. Longread deep sequencing of PCR amplicons (minus & plus 7-deaza-dGTP) reveals the need for 7-deaza-dGTP to efficiently detect expanded GAA-GGA composite alleles. IGV

pileups of longread deep sequencing of long-range PCR amplicons in the absence or presence of 7-deaza-dGTP are shown. Blue = GAA triplets; Green = GGA triplets. The fixed left-edge is the start of the GAA repeat and the variable right-edge is the site of the transposase entry into the expanded repeat (the multicolor pattern represents the adapter sequence). **(A, B)** Patient FA114 with the "single-band" pattern who is homozygous for pure GAA repeats (no composite reads are seen in either condition); **(C, D)** Patient FA64 with the "single band" pattern who is compound heterozygous for an expanded GAA-GGA composite allele and an expanded pure GAA allele (composite reads are only seen in the presence of 7-deaza-dGTP); **(E, F)** Patient FA93 with the "single-band" pattern who is compound heterozygous for an expanded GAA-GGA composite allele and an expanded pure GAA allele (composite reads are mostly, but not exclusively, seen in the presence of 7-deaza-dGTP). Black boxes indicate the location of the GGA tracks.

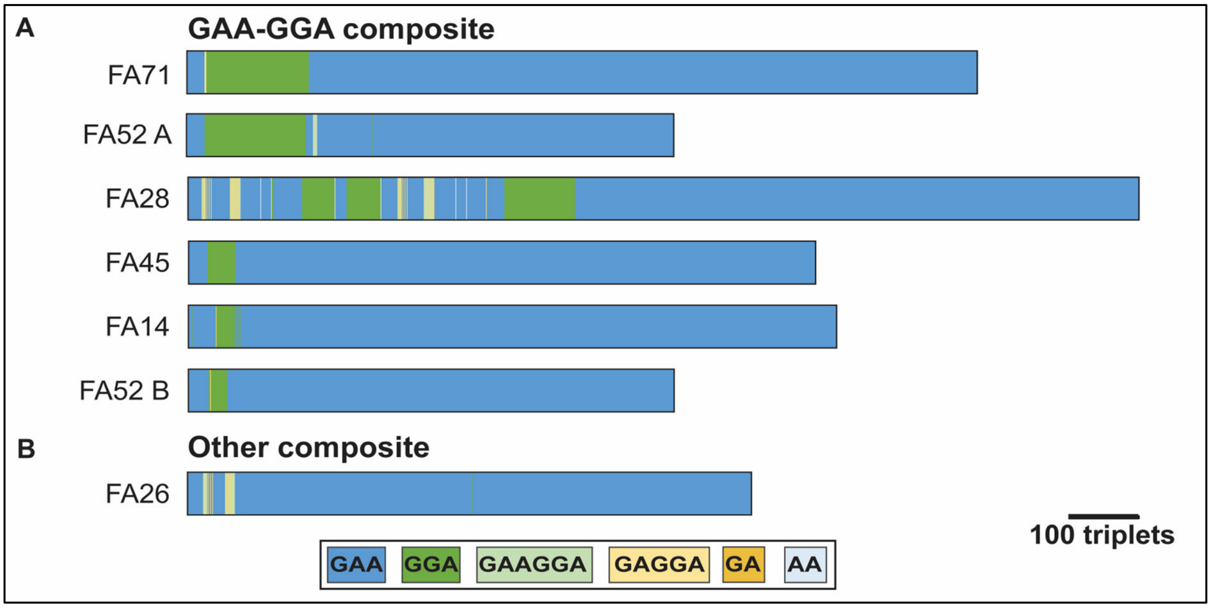

Supplementary Figure 8. Longread sequencing of PCR amplicons (plus 7-deaza-dGTP) identifies composite alleles in a validation cohort of single-band FRDA patients. (A-B) Schematic representation of (A) expanded GAA-GGA composite alleles, and (B) other composite alleles. Color-codes denote the type of non-GAA sequence, which shows a preponderance of GGA triplets (and GGA-containing hexa / pentanucleotides), that are mostly located close to the 5' end of the expanded pathogenic allele.

Supplementary Table 1. Proximal *FXN* deletions result in a severe FRDA phenotype

Patient	Genotype	Onset	Cardiomyopathy	Loss of ambulation	Scoliosis surgery	Vision loss
FA97*	Expanded GAA / Deletion	8	9	14	15	-
FA200	Expanded GAA / Deletion	1	4	6	15	-
FA199	Expanded GAA / Deletion	6	10	7	-	-
FA68	Expanded GAA / Deletion	6	6	12	12	16
FA69	Expanded GAA / Expanded GAA	17	-	-	-	-

*Deceased at 16 y

Age of onset of symptoms, cardiomyopathy, loss of ambulation, scoliosis surgery and vision loss (all in years) in the four FRDA patients confirmed to have the proximal *FXN* deletion. FA97 is unrelated to any of the others, and is compound heterozygous for the proximal *FXN* deletion and an expanded pure GAA repeat. FA200 and FA199 are sibs, and both are compound heterozygous for the proximal *FXN* deletion and an expanded pure GAA repeat. FA68 and FA69 are sibs. However, while FA68 is compound heterozygous for the proximal *FXN* deletion and an expanded pure GAA repeat, FA69 is homozygous for expanded pure GAA repeats. Note the extreme disparity in the phenotypes of FA68 and FA69. All values represent age in years; “-” indicates not applicable.

Supplementary Data 1. Supplementary Data 1.xlsx

Description and sequences of alleles in FRDA patients included in Fig. 2

Supplementary Data 2. Supplementary Data 2.xlsx

Description and sequences of alleles in FRDA patients included in Fig. 3

Supplementary Data 3. Supplementary Data 3.xlsx

Description and sequences of alleles in FRDA patients included in Supplementary Fig. 8

Supplementary Data 4. Supplementary Data 4.xlsx

Description and sequences of alleles in FRDA patients included in Fig. 6
